# Antiviral Activity of the G-Quadruplex Ligand TMPyP4 against Herpes Simplex Virus-1

**DOI:** 10.3390/v13020196

**Published:** 2021-01-28

**Authors:** Sara Artusi, Emanuela Ruggiero, Matteo Nadai, Beatrice Tosoni, Rosalba Perrone, Annalisa Ferino, Irene Zanin, Luigi Xodo, Louis Flamand, Sara N. Richter

**Affiliations:** 1 Department of Molecular Medicine, University of Padua, 35121 Padua, Italy; artusi.sara@gmail.com (S.A.); emanuela.ruggiero@unipd.it (E.R.); matteo.nadai@unipd.it (M.N.); beatrice.tosoni@studenti.unipd.it (B.T.); RPerrone@buckinstitute.org (R.P.); irene.zanin@unipd.it (I.Z.); 2 Department of Microbiology, Infectious Disease and Immunology, Faculty of Medicine, Laval University, Quebec, QC G1V 4G2, Canada; louis.flamand@crchudequebec.ulaval.ca; 3 Department of Medicine, University of Udine, 33100 Udine, Italy; annalisa.ferino@uniud.it (A.F.); luigi.xodo@uniud.it (L.X.)

**Keywords:** HSV-1, G-quadruplex, TMPyP4, TMPyP2, G-quadruplex ligands, antiviral activity

## Abstract

The herpes simplex virus 1 (HSV-1) genome is extremely rich in guanine tracts that fold into G-quadruplexes (G4s), nucleic acid secondary structures implicated in key biological functions. Viral G4s were visualized in HSV-1 infected cells, with massive virus cycle-dependent G4-formation peaking during viral DNA replication. Small molecules that specifically interact with G4s have been shown to inhibit HSV-1 DNA replication. We here investigated the antiviral activity of TMPyP4, a porphyrin known to interact with G4s. The analogue TMPyP2, with lower G4 affinity, was used as control. We showed by biophysical analysis that TMPyP4 interacts with HSV-1 G4s, and inhibits polymerase progression in vitro; in infected cells, it displayed good antiviral activity which, however, was independent of inhibition of virus DNA replication or entry. At low TMPyP4 concentration, the virus released by the cells was almost null, while inside the cell virus amounts were at control levels. TEM analysis showed that virus particles were trapped inside cytoplasmatic vesicles, which could not be ascribed to autophagy, as proven by RT-qPCR, western blot, and immunofluorescence analysis. Our data indicate a unique mechanism of action of TMPyP4 against HSV-1, and suggest the unprecedented involvement of currently unknown G4s in viral or antiviral cellular defense pathways.

## 1. Introduction

The herpes simplex virus 1 (HSV-1) is estimated to be prevalent globally in two thirds of the population less than 50 years of age [1]. It most commonly causes vesicular lesions affecting the mucous membranes; however, more serious diseases such as encephalitis, which has a reported 30% mortality rate (70–80% if left untreated), disseminated neonatal infections, and visceral infections can also occur less frequently, and especially in the immunocompromised patient [2]. HSV-1 is a neurotropic virus: after the first lytic infection within mucosal epithelial cells, the virus enters sensory neurons, where latency is established. The virus can later reactivate, resulting in the generation of new virions that cause recurrent disease [3]. HSV replication is temporally regulated and characterized by a coordinated and sequential cascade of expression of three classes of viral genes that yield immediate-early, early, and late proteins. HSV-1 symptoms are treated with nucleoside analogues, such as acyclovir (ACV) and its analogues [4]. However, the virus remains in the body for life, since no cure that eradicates herpes has yet been developed. In addition, the emergence of resistance to current anti-herpetic drugs has long created an obstacle for the treatment of HSV-1 [5]. Therefore, new antiviral approaches, able to suppress both lytic and possibly also latent infections, are required.

HSV-1 has a double-stranded linear DNA genome that is approximately 152 kbp in length, and which consists of two unique segments, surrounded by terminal inverted repeats. We have previously reported that the G-rich regions found in the terminal inverted repeats and in the promoters of the immediate early genes can fold into stable G-quadruplex (G4) structures [6,7]. In HSV-1 infected cells, G4s were shown to form massively and peak at the time of viral DNA replication [8].

G4s are DNA and RNA non-canonical nucleic acid structures that may form in G-rich regions of the organisms’ genome and transcripts. In eukaryotes, they have been proposed to exert fundamental biological roles, such as transcriptional regulation at gene promoters and enhancers, translation, chromatin epigenetic regulation, and DNA recombination [9]. Expansion of G4-forming motifs has been associated with human neurological disorders [10]. Several reports have now also addressed the presence of G4s in human viruses [11]. Due to the involvement of G4s in so many human diseases, several compounds targeting G4s have been developed. 

**Figure 1:**
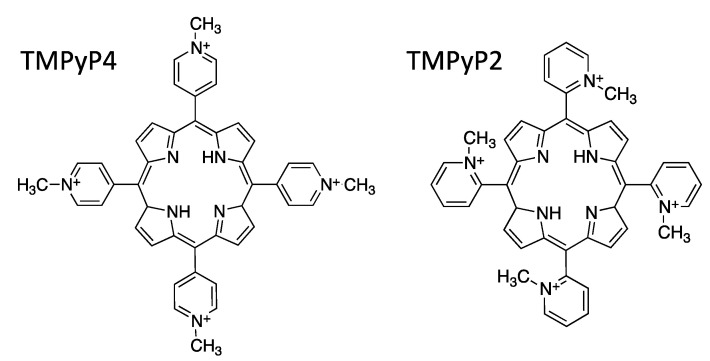
Chemical structures of the cationic porphyrin 5,10,15,20-tetra-(*N*-methyl-4-pyridyl)porphyrin (TMPyP4) and the positional isomeric cationic porphyrin 5,10,15,20-tetra-(*N*-methyl-2-pyridyl)porphyrin (TMPyP2). TMPyP4 is a G4-ligand and is the compound under study; TMPyP2 has a lower affinity for G4s, and was used here as negative control [29,30,31].

Among them, TMPyP4, a cationic porphyrin (5,10,15,20-tetra-(*N*-methyl-4-pyridyl)porphyrin; Figure 1), has been reported to stabilize G4 structures, with the consequent inhibition of telomerase activity in cancer cells [12,13,14], and modification of gene transcription levels [15,16,17,18,19,20,21,22,23,24,25,26,27,28]. This porphyrin molecule stabilizes both G4s and i-motifs, whereas its structural isomer, TMPyP2 (Figure 1), does not [29,30,31], and therefore it is used as a control to assess the specificity of TMPyP4 for G4s. 

We here investigated the activity of TMPyP4 against HSV-1.

## 2. Materials and Methods 

### 2.1. G-Quadruplex Ligands and Oligonucleotides

TMPyP4 and TMPyP2 were purchased from Calbiochem (Merck Millipore, Darmstadt, Germany) and Livchem Logistics GmbH (Frankfurt, Germany), respectively, and dissolved in sterile water.

Oligonucleotides used in this study are reported in Table 1. 

### 2.2. Circular Dichroism 

Circular dichroism spectra were recorded on a JASCO-810 spectropolarimeter and Chirascan^TM^ CD Spectrometer (Applied PhotoPhysics, UK), both equipped with a temperature controller (Peltier Jasco PTC-4235), and using a 0.5 cm-path length quartz cuvette. DNA oligonucleotides (Table 1) were diluted to a final concentration of 4 μM in a buffer containing lithium cacodylate (10 mM, pH 7.4) and 0, 0.5, 2, 20, 50, or 100 mM KCl. All samples were denatured by heating at 95 °C for 5 min, gradually cooled to room temperature, and measured after 24 h with a Nanodrop 1000 Spectrophotometer (Thermo Scientific, Illkirch Cedex, France). TMPyP4 or TMPyP2 were added after DNA folding at a final concentration of 16 μM. CD experiments were performed using the following parameters: speed scanning 50–100 nm/min, response time 4 s, accumulation 2, wavelengths from 230–320 nm or 230–600 nm in the presence of compounds. CD spectra were baseline-corrected for signal contributions due to the buffer, and the obtained ellipticity (mdeg) was converted into molar ellipticity ((θ) = deg × cm^2^ × dmol^−1^) based on sample concentration. Spectra were recorded at 20 °C, or over a temperature range of 20–90 °C with temperature increase of 5 °C. 

### 2.3. Taq-Polymerase Stop Assay

*Taq*-polymerase stop assay was initially carried out using synthesized DNA oligonucleotides (Table 1). T4 polynucleotide kinase was from Invitrogen (Paisley, UK), [γ^−32^P]ATP from Perkin Elmer (MA, USA). Primer (HSV Taq primer, Table 1) was 5′-end labelled with [γ^−32^P]ATP using T4 polynucleotide kinase at 37 °C for 30 min. The labelled primer (final concentration 72 nM) was annealed to the template (final concentration 36 nM) in lithium cacodylate buffer (10 mM, pH 7.4). Where specified, samples were incubated with KCl 10 mM in the presence or absence of TMPyP2 (500 nM) or TMPyP4 (125 nM, 250 nM, 500 nM) at room temperature. Primer extension was accomplished with 2 U of Ampli***Taq*** Gold DNA polymerase (2 U/reaction, Applied Biosystem, Carlsbad, CA, USA) at 60 °C for 30 min. Reactions were stopped by ethanol (EtOH) precipitation, primer extension products were separated on a 15% denaturing gel, and finally visualized by phosphorimaging (Typhoon FLA 9000). Markers were prepared based on the Maxam and Gilbert sequencing protocol by PCR reaction with ^32^P-labeled primer. PCR products were treated with formic acid for 5 min at room temperature, precipitated with EtOH, and then cleaved with piperidine (Sigma Aldrich, 10% solution in water) for 30 min at 90 °C [32].

### 2.4. Cell Lines and Viruses

Vero cells (African green monkey from ATCC, CCL-81) were propagated in Dulbecco’s modified Eagle’s medium (DMEM GIBCO, Life Technologies, Monza, Italy or Corning Cellgro, Manassas, VA, USA) supplemented with 10% fetal bovine serum (FBS, GIBCO Life Technologies, Monza, Italy or Sigma Aldrich, Canada), and supplemented with non-essential amino acids (Sigma-Aldrich), HEPES, sodium pyruvate (Multicell Wisent Inc., St-Bruno, QC, Canada) and plasmocin 5 µg/mL (InvivoGen, San Diego, CA, USA) or PenStrep 1X (Life Technologies). The HSV-1 strain F was kindly provided by B. Roizman (University of Chicago, Chicago, IL, USA) and ATCC (VR-733). 

### 2.5. Cytotoxicity

Cytotoxicity testing of the compounds was performed by a 3-(4,5-dimethylthiazol-2-yl)-2,5-diphenyltetrazolium bromide (MTT) assay. Vero cells were plated at a density of 1 × 10^4^ cells/well in 96-well tissue culture plates to a final volume of 100 µL, and were grown overnight. To the cells were next added increasing concentrations of TMPyP4 or TMPyP2 previously dissolved in water. Each sample was tested in triplicate. At 48 h post treatment cell survival was evaluated: a freshly dissolved MTT solution (5 mg/mL in sterile PBS 1X, Sigma Aldrich) was added and cells were incubated for 4 h at 37 °C, CO_2_ 5%. Then, 100 µL/well of SDS-HCl solution (sodium dodecyl sulfate 10%, HCl 0.01 M) was then added and cells were incubated overnight at 37 °C. Absorbance was determined at 620 nm (Sunrise Tecan Spectrophotometer). Cell growth was also monitored by count: Vero cells were plated and treated with increasing compound concentrations; the compound was refreshed every 24 h. Cell growth was assessed by counting cell number every 24 h (Cellometer Auto T4, Nexcelom Bioscience).

### 2.6. Flow Cytometry

Vero cells were seeded in 6-well, 1.8 × 10^5^ cells per well in DMEM + FBS10%, and treated after 16 h with TMPyP4 1 μM. At indicated time points post treatment, cells were collected in a FACS tube, washed with PBS 1X, and fixed in cold EtOH 70% at 4 °C. Then, cells were washed with PBS 1X, treated with RNase at 37 °C for 30 min. PI was added prior to analysis, which was performed on a FACS Cytofluorometer BD LSR II (BD Bioscences, NJ, USA). Fluorescence acquisitions were analyzed with FlowJo^TM^ software (Tree Star, OR, USA).

### 2.7. Viral Titration Assay

Vero cells were seeded at a density of 2.5 × 10^5^ cells per well in 6-well tissue culture plates, pre-treated with TMPyP4 or TMPyP2 (0.04 µM–25 µM) for 16 h, infected with HSV-1 strain F at a multiplicity of infection (MOI) of 1 plaque forming units (PFUs)/cell. After 1 h infection at 37 °C, cells were washed and maintained in culture medium supplemented with TMPyP4, TMPyP2 (0.04 µM–25 µM), or DMEM/FBS 10% as a non-treated control. At 24 h post infection (h.p.i.), supernatants were collected and titrated. Virus plaque assay was performed using confluent Vero cells in 24-well culture plates (1 × 10^5^ cells/well). Cells were infected with 250 µL of serially diluted (10-fold) supernatants for 1 h at 37 °C, every dilution was tested in triplicate. After the infection, cells were washed and incubated with 500 µL of DMEM supplemented with 2% FBS and 0.6% methyl cellulose (Sigma-Aldrich). Viral plaques were counted at 48 h.p.i. by fixing the cells’ monolayer with 1 mL/well of formaldehyde 5% and crystal violet 0.8% (in ethanol 50%). EC_50_ values were calculated according to standard dose–response curve analysis.

### 2.8. Early-Entry Viral Assay

Vero cells were seeded in a 24-well plate at a density of 1 × 10^5^ cells per well tissue culture plates. To assess whether TMPyP4 had an impact on viral entry or fusogenic events, cells were treated at various time points (at −1, 0, and 2 h relative to infection) with TMPyP4 or TMPyP2 (1 µM) and infected with HSV-1 strain F, MOI of 1 PFU/cell. One h after infection, cells were washed with PBS 1X and maintained at 37 °C in culture medium supplemented with the drug. At 30 h.p.i. the amount of infective viral particles in the supernatants was analyzed. 

### 2.9. Quantitative Polymerase Chain Reaction (q-PCR)

Total HSV-1 intracellular DNA was collected from confluent Vero cells, similarly to the procedure used to collect viral supernatants described above. At 3 or 20 h.p.i., cells were collected and DNA was extracted using a QIAamp DNA blood Mini Kit (QIAgen), according to the manufacturer’s instructions. Forward/reverse primers used to perform q-PCR (Sigma) were designed within conserved HSV-1 gene sequences, using Primer Express 3 (Applied Biosystem) (Table 1). HSV-1 intracellular DNAs were analyzed by q-PCR using SYBR^®^ green as the detection reagent. The reaction was performed in a final volume of 20 µL containing SYBR^®^ green fast 2X (QIAgen), forward and reverse primers at 500 nM, and isolated viral DNA at 100 nM. Data were collected using a Rotor Gene Q thermo cycler (QIAgen) with the following cycling conditions: 95 °C for 5 min, followed by 40 cycles of 5 s at 95 °C, and 10 s at 60 °C.

### 2.10. TMPyP4 Localization in Cells

Vero cells (kidney epithelial cells from an African green monkey) were plated (2.5 × 10^5^ cells/dish) on coverslips placed in 35 mm Petri dishes. When the cells reached 60–70% confluence they were treated either with 5, 10, or 25 µM TMPyP4 for 20 h in the dark. Then cells were washed twice with PBS and fixed with 3% paraformaldehyde (PFA) in PBS for 20 min. After quenching with 0.1 M glycine solution containing 0.02% sodium azide in PBS, and permeabilization with Triton X-100 (0.1%) in PBS, the coverslips were incubated for 5 min with Hoechst to stain the nuclei, and mounted with Mowiol^®^ (Sigma Aldrich). Images were obtained with a Leica TCS SP8 confocal system (Leica Microsystems GmbH, Germany), equipped with a 405 nm diode laser and a pulsed super-continuum white light laser, using a 63x/11.40 oil immersion objective (Hoechst: ex = 405 nm, detection range = 415–465 nm, PMT detector; TMPyP4: ex = 580 nm, detection range = 600–783 nm, HyD detector).

### 2.11. Transmission Electron Microscopy (TEM)

Vero cells were infected with HSV-1 strain F and treated with TMPyP4 or TMPyP2 (1, 5, 25 µM), as described above. At 24 h.p.i., cells were washed twice with PBS 1X, detached with trypsin, and collected by centrifugation at 300 rcf for 5 min at room temperature. Pellets were carefully resuspended with 2.5% glutaraldehyde (Sigma Aldrich) in PBS 1X and incubated at room temperature for 4 h, then stored at 4 °C. Cells were processed for electron microscopy analysis (FEI Tecnai™), and sections were collected on nickel grids according to standard protocols. 

### 2.12. Western Blot

Vero cells were pre-treated for 16 h with TMPyP4 or complete DMEM and 1% water, then infected with HSV-1 at a MOI of 1 for 1 h at 37 °C, and maintained with or without TMPyP4 for 24 h. Cells were harvested and lysed in RIPA 1X buffer containing protease inhibitors (Roche). Lysates were quantified and resolved by 10% SDS/PAGE and transferred through semi-dry transfer for 19 min at 24 V. Membrane was blocked with 5% milk in PBS-T (0.05% Tween-20 (Sigma Aldrich) in PBS 1X) for 1 h at room temperature and incubated with the primary antibodies p62 (Sigma Aldrich) at 1:500 overnight at 4 °C. Blot was subsequently incubated with anti-rabbit CYP5 antibody 1:2500 for 1 h at room temperature, and analyzed on a Thyphon FLA 9000.

### 2.13. Immunofluorescence

Vero cells were seeded at 5 × 10^4^ cells/well on glass 12-mm-diameter coverslips in 24-well plates and grown overnight at 37 °C. Following 16 h pre-treatment with TMPyP4 (1 µM), cells were infected with HSV-1 v41 [33] at a MOI of 1 for 1 h at 37 °C, maintained in culture with or without TMPyP4 (1 μM) for 24 h.p.i., and then fixed in 4% paraformaldehyde (PFA, Sigma Aldrich) for 20 min at room temperature. Cells were permeabilized with 0.5% Tween-20 (Sigma Aldrich) in PBS 1X, blocked in PBS containing FBS 5%, and incubated with an anti-p62 antibody (Sigma Aldrich) at 1:1000 for 1 h at room temperature. Cells were then incubated with Alexa Fluor-546 secondary antibody at 1:500 (Molecular Probes, Life Technologies) for 1 h, and nuclei were stained with DRAQ5^®^, 1:1000 (Cell Signaling Technology, Danvers, MA, USA) for 5 min. Imaging was performed with a Nikon A1Rsi + Laser Scanning confocal microscope, equipped with NIS-Elements Advanced Research software (Nikon Instruments Inc., Melville, NY, USA), and with 20× and 60× ocular objectives.

### 2.14. RT-qPCR

Vero cells were seeded in 6-well plates at a density of 1.8 × 10^5^ cells per well and incubated overnight. The following day, cells were pre-treated with TMPyP4 at a final concentration of 1 µM for 16 h. At the end of incubation, cells were infected with HSV-1 strain F for 1 h at a MOI of 1. Treatment with 1 µM TMPyP4 was repeated, and cells were collected at 5, 8, 15, and 24 h.p.i. Total RNA was isolated using a GeneJET RNA Purification Kit (ThermoFisher), according to the manufacturer’s instructions, and subjected to RNase free DNase I treatment (ThermoFisher) to remove residual genomic DNA. The concentration of the harvested RNA was measured with a NanoDrop 1000 spectrophotometer (ThermoFisher). The required amount of RNA (500 ng) was reverse transcribed into cDNA using MultiScribe Reverse Transcriptase (ThermoFisher) following the manufacturer’s instructions. The obtained cDNA was subjected to RT-qPCR using SYBR Green PCR Master Mix 2X (ThermoFisher) with 150 nM primers. Experiments were performed using a LightCycler 480 System (Roche) under the following conditions: one cycle of 10 minutes at 95 °C followed by 40 cycles of 1 min at 95 °C, followed by 30 s at 60 °C, and 30 s at 72 °C. Each sample was analyzed in duplicate. The Ct values were normalized to the genomic mean of the housekeeping gene *ACTB*, and the relative gene expression was determined using the Livak method, 2^−ΔΔ*C*t^. 

## 3. Results

### 3.1. The G4-Ligand TMPyP4 Does Not Impair the Early Stages of the HSV-1 Life Cycle

We have previously shown that G4 ligands inhibit HSV-1 replication by interacting with multiple G4-forming sequences present in the repeated regions of the viral genome [6,34], thus impairing viral replication. In this line of research, we tested the G4 ligand TMPyP4 and its analogue TMPyP2, which is reported to have a lower affinity for G4s, and thus is normally used as a negative control compound when testing TMPyP4 activity [35,36]. 

**Figure 2:**
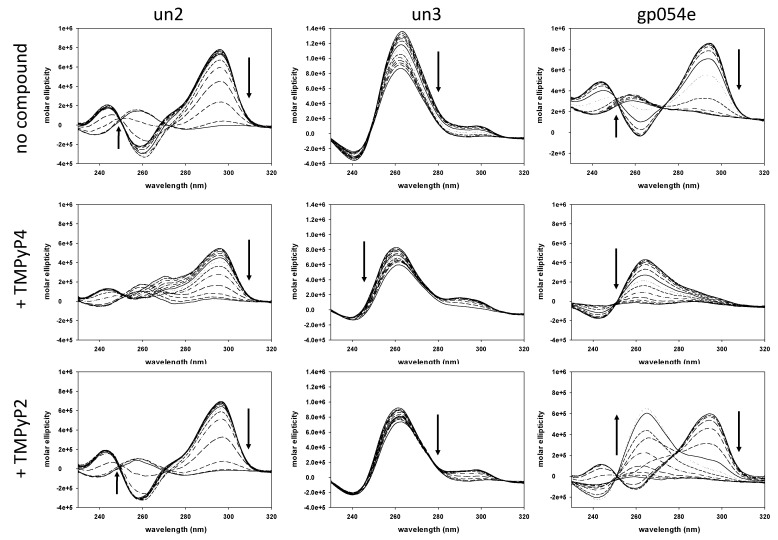
Thermal unfolding spectra of three HSV-1 G4-forming sequences, *un2*, *un3*, and *gp054e* (4 μM), alone or in the presence of TMPyP4 or TMPyP2 (16 μM). Arrows in circular dichroism (CD) spectra indicate direction of the spectra upon temperature increase/DNA denaturation. Spectra were recorded over a temperature range of 20–90 °C.

We first studied if TMPyP4 could interact with the identified HSV-1 G4 forming sequences (Table 1), and compared its activity to that of TMPyP2 by circular dichroism (CD). As previously reported, at physiological concentrations of K^+^ 100 mM all tested oligonucleotides displayed a melting temperature (T_m_) above 95 °C; we thus lowered K^+^ concentration to obtain measurable and comparable T_m_ values among the oligonucleotides. In these conditions all three sequences displayed CD signatures characteristic of G4 conformations: mainly antiparallel (*un2* and *gp054e*) and parallel (*un3*) [6]. Upon incubation with TMPyP4, the overall CD signature did not change for the *un3* sequence, while it was converted from antiparallel to hybrid in the case of the *un2* and *gp054e* sequence. The molar ellipticity signal generally decreased, while T_m_ values varied in a range of +1 to −9 °C, suggesting a slight destabilizing effect imparted by the G4 ligand on these sequences. Spectra and T_m_ variations were less pronounced in the presence of TMPyP2 (Figure 2).

**Figure 3:**
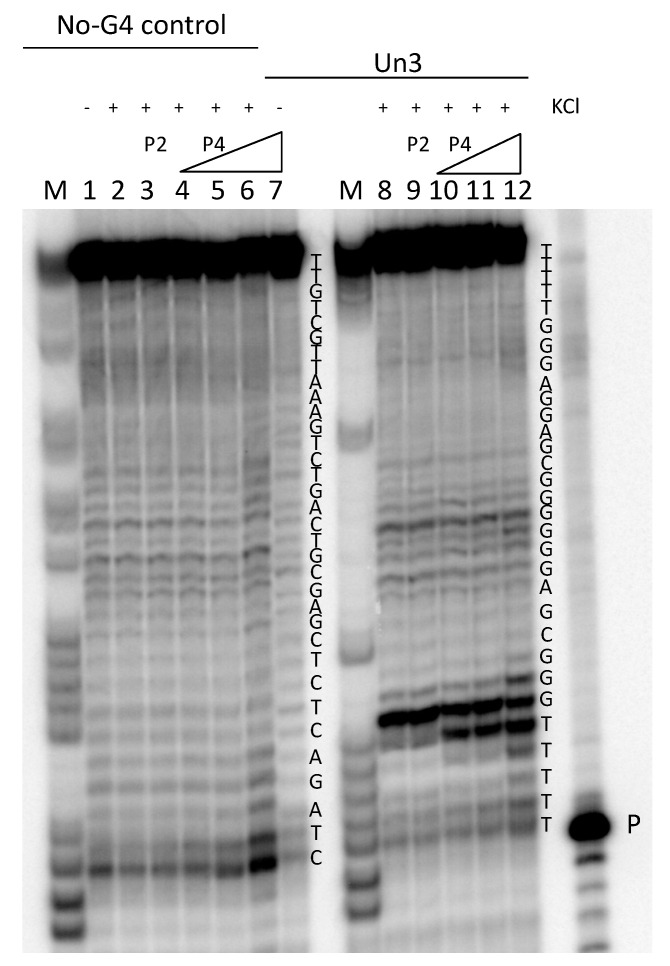
Representative *Taq* polymerase stop gel. Oligonucleotides were folded in the presence or absence of K^+^. K^+^-treated samples were further incubated with increasing concentrations (125 nM, 250 nM, and 500 nM) of TMPyP4 (P4) or the control compound TMPyP2 (P2) (500 nM). Oligonucleotides were used as templates in a *Taq* polymerase reaction at 60 °C. The G4-forming oligonucleotide *un3*, and an oligonucleotide that does not form G4 (no-G4 control), were employed. Oligonucleotide sequences are indicated on the right of each set of samples. P indicates the band of the labeled primer. M is a marker lane obtained with the Maxam and Gilbert sequencing protocol.

To assess if HSV-1 G4s could also form in a longer DNA sequence, and whether TMPyP4 had any effect on this different environment, we performed a *Taq* polymerase stop assay. We tested an extended *un3* sequence: when the oligonucleotide was folded in the presence of K^+^, a strong pausing site at the first G-tract encountered by the enzyme was observed (Figure 3, lane 8). In the presence of increasing concentrations of TMPyP4, one/two additional bands at the stop site indicated an increased stabilization of the structure (15–33% compared to the untreated control) (Figure 3, lanes 10–12), whereas TMPyP2 did not show any different effects with respect to the untreated oligonucleotide (Figure 3, compare lanes 9 and 8). Both compounds did not show activity on a sequence unable to fold into G4 (Figure 3, lanes 1–7).

Both CD and stop assay data indicated that TMPyP4 is able to interact with HSV-1 G4-folded sequences more effectively than TMPyP2.

**Figure 4:**
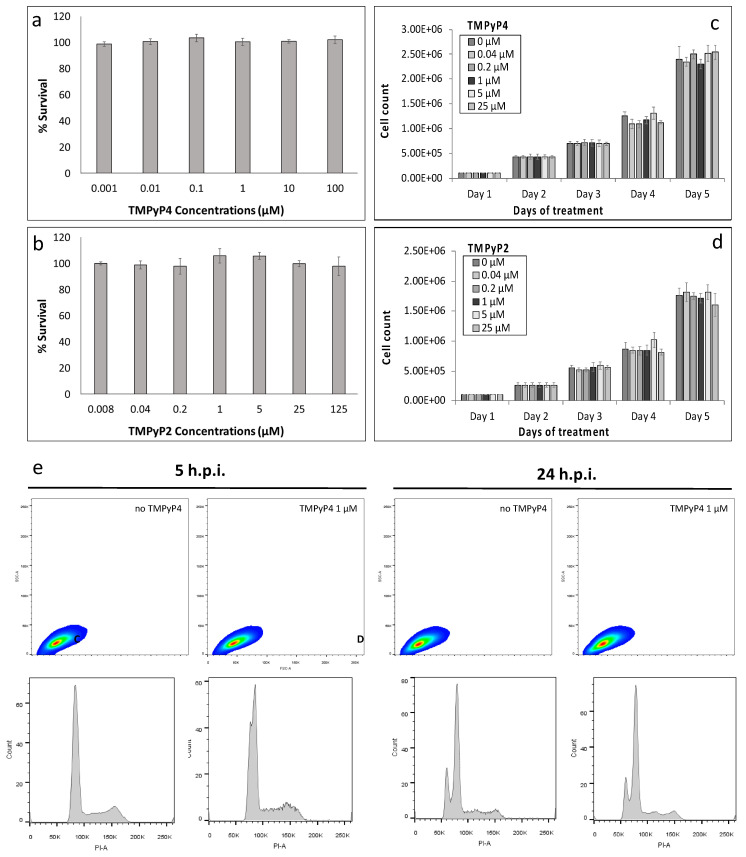
Effect of TMPyP4 on uninfected Vero cells. (**a**) Percentage of cell survival determined by MTT following treatment with TMPyP4 (**a**) or TMPyP2 (**b**). For cell count, cells were treated with either TMPyP4 (**c**) or TMPyP2 (**d**), and counted every 24 h, for a total of five days. Two replicates per analysis were performed. (**e**) Flow cytometry analysis of Vero cells in the presence of TMPyP4. Vero cells gated for granularity (SSC) and size (FSC) and relative cell cycle histograms. Analysis was performed at 5 h and 24 h post treatment, as indicated, in the absence and presence of TMPyP4 1 μM.

To assess the role of TMPyP4 and TMPyP2 against the virus, we first evaluated their toxicity profiles. Both compounds were not cytotoxic even at the highest tested dose (CC_50_ > 100 μM) and, upon daily treatment, did not impair cell growth up to day 5 (Figure 4a–d). Flow cytometry analysis also showed no cell morphology or phase variation, both at short (5 h) and long times (24 h) of exposure to TMPyP4 (Figure 4e). 

**Figure 5:**
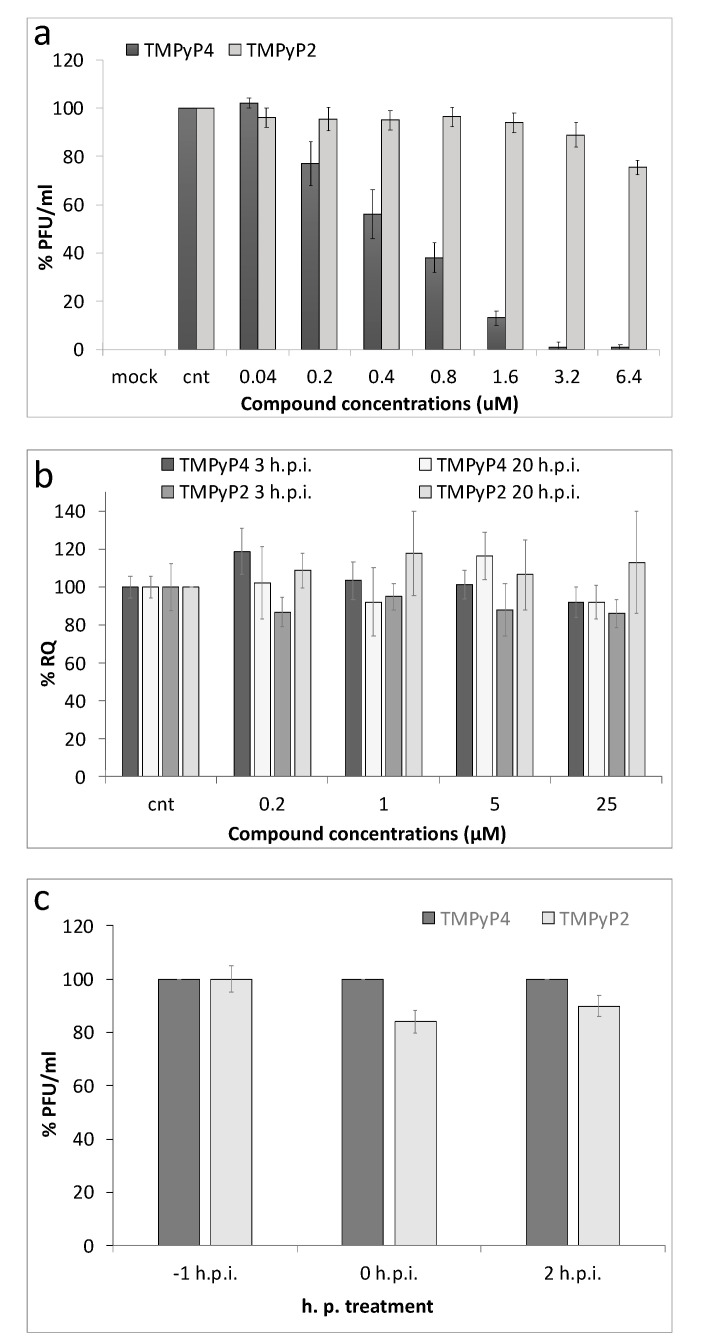
Activity of TMPyP4 in HSV-1 infected cells. (**a**) Antiviral activity of TMPyP4 determined through plaque assay: HSV-1 infected cells (multiplicity of infection (MOI) 1 plaque forming units (PFU)/cell) were treated with increasing concentrations (0.04 µM–6.4 µM) of TMPyP4 or TMPyP2, used as a negative control; supernatants were collected 24 h.p.i., and the number of PFU was determined; (**b**) Levels of intracellular HSV-1 DNA, extracted from infected Vero cells treated with the test compounds. Quantification of intracellular DNA amounts was obtained from infected cells at 3 h and 20 h.p.i., treated with increasing concentration of TMPyP4 and TMPyP2. RQ is relative quantities; (**c**) Effect of TMPyP4 and TMPyP2 (1 µM) on virus entry into the cells. Cells were treated at various time points (−1, 0, and 2 h) relative to infection with HSV-1 strain F at a MOI of 1. One h after infection, cells were washed and maintained in culture medium supplemented with the drug. At 30 h.p.i. supernatants were collected and titrated by plaque assay. Virus yields are given as % relative to the 0 h.p.i. TMPyP4-treated sample.

We next assessed through plaque assay if TMPyP4 displayed antiviral potency, similarly to other G4-ligands, such as BRACO-19 and NDIs [6,34]. Almost no infectious particles were present in the supernatant of cells treated with TMPyP4 at concentrations higher than 1 μM (EC_50_ = 500 nM) with a 90–98% antiviral activity at this dose, whereas TMPyP2 afforded minor inhibition of release of infectious particles in the supernatant, and only at the highest tested concentration, as also previously reported (EC_50_ > 25 μM) (Figure 5a) [6]. 

Following the ability of TMPyP4 to inhibit *Taq* DNA polymerase in vitro by G4 interaction, we assessed TMPyP4 effect on viral DNA replication in infected cells. HSV-1 infected cells were treated with increasing concentration of TMPyP4 (0.04–25 µM), and intracellular DNA levels were determined. Intracellular viral DNA was extracted both before (3 h.p.i.) and after (20 h.p.i.) viral DNA replication had occurred in cells, and quantified by qPCR. In comparison to the non-treated infected cells used as control, no decrease of viral DNA was detected upon TMPyP4 treatment both before and after replication (Figure 5b), indicating that TMPyP4 does not affect viral DNA replication. TMPyP2 was again used as a control, and did not impair viral DNAs at the investigated time points. 

We next tested whether TMPyP4 affected viral entry: HSV-1 infected cells were treated with the compound (5 µM) at different times: 1 h pre-infection, at infection, and up to 2 h.p.i., the time at which virus fusion to, and entry in, the cell were completed [37]. No difference in virus production tested at 30 h.p.i. was observed in these conditions (Figure 5c), indicating that TMPyP4 does not affect the attachment of the virus to the host cells. 

**Figure 6:**
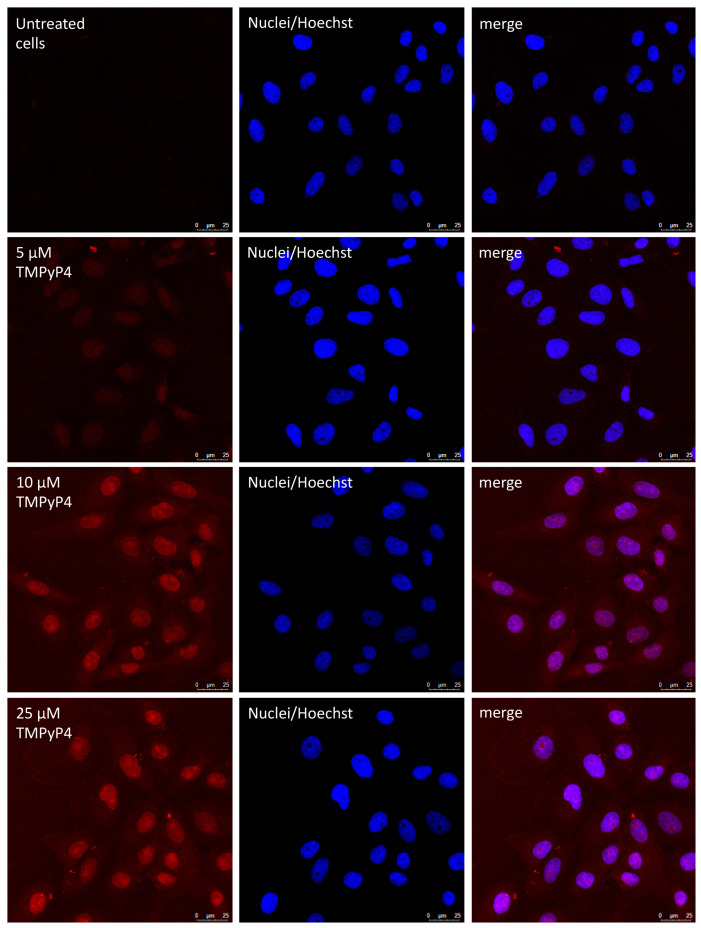
Confocal microscopy images of Vero cells untreated or treated with 5, 10, and 25 μM TMPyP4 for 20 h. Cell nuclei were stained with Hoechst. The images show that the cationic porphyrin mainly localized in the cell nucleus.

Since G4 can also form at the transcript level, and because of the discrepancy between the in vitro and in cell data, i.e., TMPyP4 inhibition of polymerase activity in vitro but not in cells, we took advantage of the fluorescence properties of TMPyP4, and tested cell entry and localization of TMPyP4. The fluorescence emission intensity allowed testing compound concentrations of 5 µM or higher (Figure 6). In these conditions, TMPyP4 mainly localized in the nucleus of cells, but a lower amount was also present in the cytoplasm.

### 3.2. TMPyP4 Induces Trapping of Fully Infectious HSV-1 Virions in Vesicles in Cells

**Figure 7:**
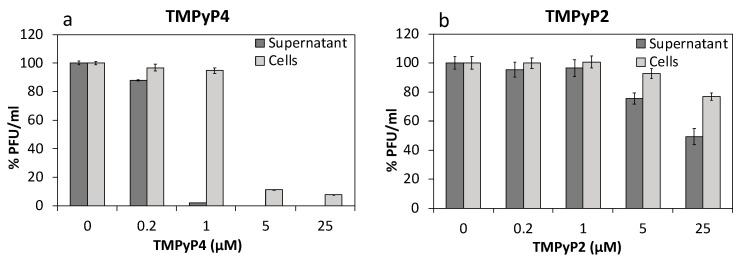
Levels of infective virions were determined through plaque assay, both in supernatants and in cell lysates at 24 h.p.i., upon treatment with TMPyP4 (**a**), and with TMPyP2 (**b**) used as negative control.

Based on the above results, we checked the later events of the virus life cycle, and thus tested whether new virus particles were produced in the cells. To this end, we assessed the presence of infective particles both in supernatants (i.e., the virus naturally released by the cell) and in cell lysates (i.e., the mature virus in the cell cytoplasm before egress from the cell) at 24 h.p.i. upon treatment with TMPyP4, and TMPyP2 as negative control. Surprisingly, at 1 µM, while infective particles in the supernatant were almost absent (as also previously observed, see Figure 5), the intracellular HSV-1 levels were approximately equal to those of untreated cells (Figure 7a). At higher TMPyP4 concentrations, however, both the intracellular and extracellular virus drastically decreased. Treatment with TMPyP2 induced much milder inhibition on both the intracellular and extracellular virus and at a compound concentration >5 µM; also in this case, the compound’s effect on the extracellular virus was greater (Figure 7b). These data suggest that TMPyP4 (and in part TMPyP2 at higher concentration) induce trapping of fully infectious HSV-1 particles in the cell cytoplasm. 

**Figure 8:**
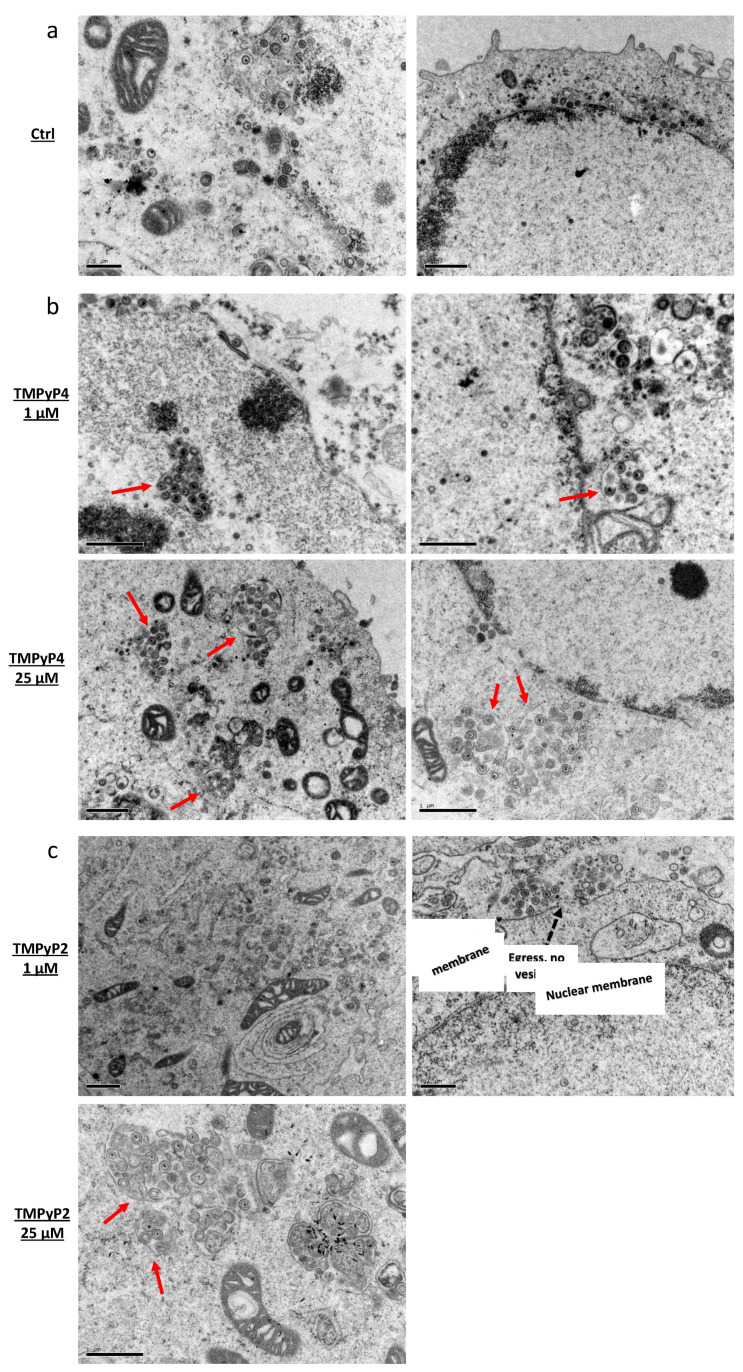
Transmission electron microscopy analysis of HSV-1 infected cells at a MOI of 1, in the absence or presence of TMPyP4 or TMPyP2. Panels display: (**a**) non-treated infected Vero cells; (**b**) infected cells treated with TMPyP4 at 1 µM (top) or 25 µM (bottom); (**c**) infected cells treated with TMPyP2 at 1 µM (top) or 25 µM (bottom). In the cytoplasm of infected/treated cells HSV-1-containing vesicles are highlighted with solid red arrows. Dashed black arrows indicate viral egress from the cytoplasmatic membrane. Black bars at the bottom left corner of each figure indicate the image scale.

To analyze the intracellular localization of HSV-1 particles upon treatment with TMPyP4 1 µM and TMPyP2 25 µM, i.e., at the concentrations where a bias between intracellular and extracellular virions amount was found, we performed transmission electron microscopy (TEM) experiments. In the TMPyP4-treated samples, we observed large vesicles that enclosed groups of HSV-1 particles in the cell cytoplasm (Figure 8b). These vesicles were characterized by their own membrane, were located close to the nuclear membrane, and were absent in the non-treated infected sample (Figure 8a). Interestingly, most HSV-1 particles appeared properly assembled, with a darker area in the center corresponding to the DNA, the capsid, and external layers that were also present in the virions forming in the untreated cells (Figure 8a,b), and which we have previously observed in HSV-1 producing cells [8]. No increase in the number of vesicles in the cytoplasm, other than those that enclosed virus particles, was detected. Treatment with TMPyP2 induced no visible vesicles at 1 µM, whereas some vesicles were observed at 25 µM (Figure 8c). These data suggest a possible role of TMPyP4 in HSV-1 maturation and/or egress [38].

### 3.3. TMPyP4-Induced Vesicles Are Independent of Autophagy

**Figure 9:**
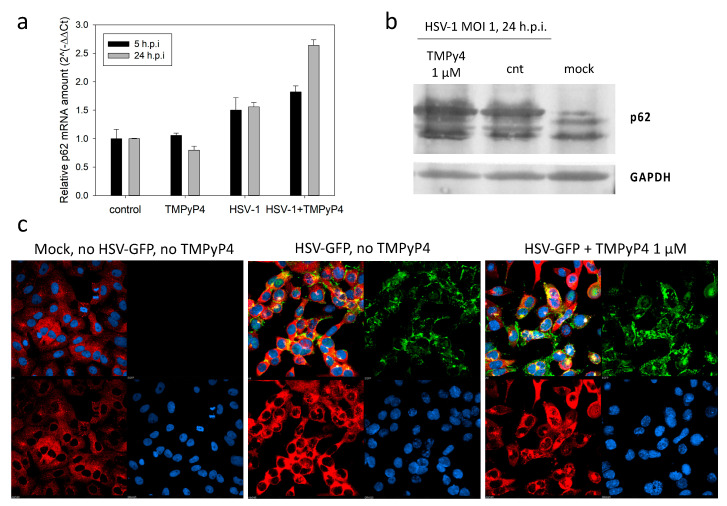
Expression of p62 upon treatment with TMPyP4. (**a**) p62 mRNA levels were analyzed by qRT-PCR. Uninfected and untreated cells were used as controls. Two replicates were performed. Standard deviation is shown. (**b**) p62 protein levels were determined through western blotting from lysates of Vero cells infected with HSV-1 at a of MOI of 1, in the absence or presence of TMPyP4 (1 μM). From right to left, lanes represent non-infected/non-treated Vero cells only, infected/non-treated Vero cells, infected/TMPyP4-treated cells. The housekeeping GAPDH (bottom lane) was used as a loading control. Three replicates were performed. (**c**) For immunofluorescence studies, Vero cells were infected with a GFP-VP16 HSV-1 (HSV-1 v41) in the absence (central panel) or presence of the G4-ligand TMPyP4 (1 μM, right panel). Mock Vero cells are shown on the left panel. Following fixation, permeabilization, and blocking, cells were probed with anti-p62 primary antibody and Alexa-546 secondary antibody. The green signal corresponds to the viral protein VP16-GFP, the red signal indicates p62, the blue signal indicates cell nuclei. Three replicates were performed.

We reasoned that the observed vesicles could be autophagy-induced lysosomes. Autophagy is an evolutionarily conserved cellular defense mechanism, in which the cell packages cytosolic constituents in double-membrane vesicles and delivers them to the lysosome for degradation [39]. HSV-1 has been reported to block formation of autophagosomes through multiple viral/cellular protein interactions [40,41]. We reasoned that TMPyP4 could counteract the virus-induced inhibition of autophagy, and therefore stimulate this process. To test this hypothesis, we evaluated the level of p62, an autophagic flux marker: p62 accumulates when autophagy is inhibited, and vice versa it decreases when autophagy is induced [42]. When the levels of p62 were analyzed by RT-qPCR and Western blot (Figure 9a,b, respectively), we detected an increase of p62 in the untreated HSV-1 infected cells, data that confirm the reported inhibition of autophagy by the virus [42]. Upon treatment with 1 µM TMPyP4, a further increase in p62 mRNA and protein levels was appreciable (Figure 9a,b, respectively). We next tested p62 in cells by immunofluorescence (Figure 9c): in this case we used HSV-1 v41, a recombinant virus expressing the viral protein VP16 fused to the green fluorescent protein (GFP), so that we could visualize the presence of the virus in the cells. This mutant virus is characterized by normal replication kinetics and yields [33].

This concentration of TMPyP4 did not appreciably alter the intensity or distribution of GFP-VP16 within non-treated versus treated cells (Figure 9b). The p62 signal was almost absent in uninfected cells, and it became visible in HSV-1 infected cells; upon treatment with TMPyP4, we did not detect a significant variation in p62 levels (Figure 9c). These data indicate that the vesicles observed in TEM analysis when TMPyP4 was used do not share the autophagy pathway. 

**Figure 10:**
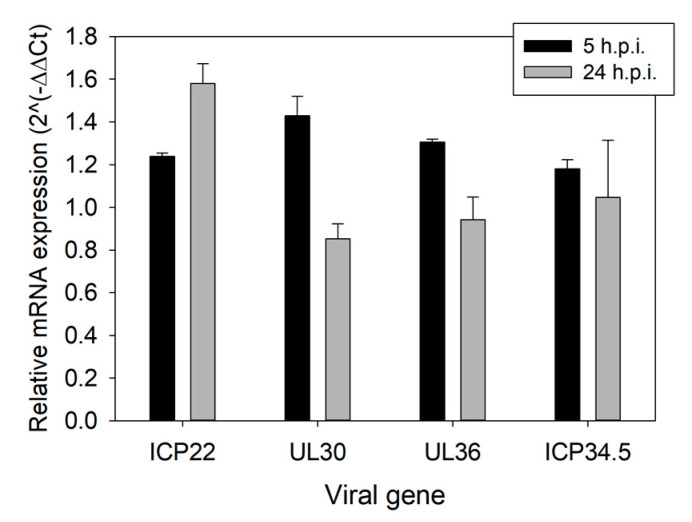
RT-qPCR analysis of mRNA of the viral genes that express UL36, UL30, ICP22, and ICP34.5. Relative viral mRNA expression upon treatment with TMPyP4 (1 µM) is reported relative to the same sample without treatment (value of 1). Thus, samples that have relative mRNA expression higher and lower than 1 have higher and lower mRNA expression relative to their untreated control. All experiments were performed at least twice. Standard deviation is shown in all analyses.

We next assessed TMPyP4’s effect on four unrelated viral genes that express proteins involved in different steps of the viral life cycle: the immediate-early ICP22, a transcriptional regulator of cellular and viral mRNAs; the early UL30, the DNA polymerase catalytic subunit; the late UL36, the large tegument protein, in the coding sequence of which the *gp054e* G4 sequence is present [6]; the leaky late ICP34.5, which, besides many other activities, antagonizes the host autophagy response, and the promoter of which contains the *un3* G4 sequence [6]. At 5 h.p.i., expression of all proteins treated with TMPyP4 was mildly stimulated compared to untreated infected cells; however, at 24 h.p.i. only ICP22 was further stimulated, while UL30 was downmodulated, and UL36 and ICP34.5 showed no variation compared to untreated infected cells (Figure 10). These data suggest that TMPyP4 at 1 µM exerts its activity at different levels during the viral life cycle.

## 4. Discussion

We have shown here the unique antiviral activity of the G4-ligand TMPyP4 against HSV-1. The antiviral EC_50_ was 3–30 times lower than that obtained to inhibit telomerase activity [13,43], as also proved by the absence of cytotoxicity in the concentration range necessary to observe complete antiviral activity. Like previously reported G4-ligands [6,34], TMPyP4 was able to interact and stabilize the most abundant HSV-1 G4-forming sequences found in the repeat regions of the viral genome. However, differently from the other compounds, TMPyP4 did not impair viral replication in cells. The reason for this different behavior could be at multiple levels: (i) TMPyP4 could preferentially stabilize viral G4s that have not yet been characterized. HSV-1 has one of the most G-rich and long genomes among viruses, with the theoretical possibility of forming hundreds of DNA G4s, as recently predicted by a computational approach [44]; in addition, non-canonical G4s have also been shown to be present and function in cells, therefore the total number of HSV-1 DNA G4s may largely exceed the predicted one. We have recently shown that the promoters of all HSV-1 immediate early genes contain conserved G4-forming sequences [7], thus highlighting the possible role of G4 structures at specific steps of the viral cycle other than DNA replication. Given these considerations, one possibility is that TMPyP4 best-affinity DNA G4 target is yet to be described and characterized; (ii) G4s also form at the RNA level, and we showed that a fraction of the intracellular compound also localizes in the cell cytoplasm; indeed, TMPyP4 has been reported to destabilize cellular RNA G4s with, in some cases, therapeutic implications [45,46,47]. The number of HSV-1 RNA G4s has not yet been assessed; however, considering the higher stability of RNA versus DNA G4s of the same sequence [48], thousands of structures are also expected in the case of viral RNA G4s. One or more of these could also be the preferential target/s of TMPyP4, with an unanticipated mechanism of action. In addition, the unusual destabilizing activity on RNA G4s may exert unexpected biological effects; (iii) Among G4-ligands, TMPyP4 has one of the lowest G4-specificities, having also been described to bind other nucleic acid secondary structures. One of these is the i-motif conformation, which may form in C-rich sequences, typically as counterparts of the G-rich G4-forming regions [49,50], and which TMPyP4 has been reported to bind to [51,52,53,54,55]. In this case, as well, no indication of HSV-1 i-motifs has yet been reported, but stabilization of one or more of these structures may be responsible for the observed peculiar antiviral activity of TMPyP4; (iv) In line with the ability of the compound to bind different nucleic acid structures, the double stranded DNA may also be recognized [56,57,58], and activity at this level opens up several additional scenarios as for the mechanism of the drug. 

TMPyP4 has been previously investigated by us and other groups on several other viruses. In the HIV-1 virus, TMPyP4 inhibited *nef* gene expression by stabilizing a G4 cluster in the *nef* gene coding region, and it inhibited viral infectivity in a dose dependent manner in a concentration range similar to the one used here (0.1–6 µM). TMPyP2 was used as a control drug [59]. In the Epstein–Barr virus (EBV) TMPyP4, used at 10 µM, interfered with recruitment of the origin recognition complex by the nuclear viral protein EBNA1. The G4-mediated mechanism of action was gathered by comparison with TMPyP2 [60]. In Kaposi sarcoma herpes virus (KSHV), TMPyP4 was shown to stabilize a G4 present in the mRNA of the latency-associated nuclear protein LANA, and thus inhibit protein translation. This effect in cells was reached at a TMPyP4 concentration of 10 µM [61]. In HCV TMPyP4 was reported to bind the G4 sequences that can form in the viral genome: this activity resulted in inhibition of RNA-dependent RNA polymerase, of gene expression, and of HCV replication in cells. The tested concentrations were higher than those at which we observed vesicle formation in HSV-1 (1.25–10 µM). The observed effect was ascribed to a G4-mediated mechanism of action, because of the lower effects observed with the analogue TMPyP2 [62]. In Ebola virus, TMPyP4 inhibited gene expression and intracellular replication of an artificial Zaire ebolavirus mini-genome in a G4-mediated mechanism on the viral RNA. In this case the compound was studied in a concentration range that comprises the one at which we showed vesicle formation (0.25–10.00 µM) [63]. Interestingly, in all viruses but HIV-1, TMPyP4 activity was evaluated at the RNA level, even in EBV, which belongs to the Herpesviridae family, like HSV-1; in all cases no effect on trafficking was investigated nor reported. However, as viruses are so diverse in their genomes, life cycles, and interactions with the host cell, a direct comparison of TMPyP4’s effect (and their trafficking mechanism) is hardly meaningful.

We believe that the TMPyP4 activity observed in HSV-1 derives from the engagement of more than one of the above-described possible targets: at 1 µM one of the targets prevails over the others, and induces virion retention in cellular cytoplasmatic vesicles. We expect this peculiar and unique behavior to be the result of the interaction of TMPyP4 with a specific G4 target, as proven by the essentially lower effect of TMPyP2, which parallels the compound’s lower G4 affinity [35,36]. At higher TMPyP4 concentration, different target/s may also become engaged [64]; because of the absence of cytotoxicity in the tested conditions, specific viral targets are still likely involved; however, an interaction between TMPyP4-stimulated viral effectors and cellular components may also be present.

Even if the identification of TMPyP4 target/s will require a much deeper and intensive investigation, the present data indicate a unique mechanism of action of TMPyP4 against HSV-1, and suggest the unprecedented involvement of currently unknown G4s in viral or antiviral cellular defense pathways. Acquisition of this TMPyP4 unique mechanism of action will likely lead to the discovery of as yet unidentified mechanisms of viral infection and virus/cell interactions. 

## Figures and Tables

**Table 1:** Oligonucleotides used in this study.

Assay	Name	Sequence (5′→3′) *
CD	gp054e	**GGGG**CT**GGGG**CT**GGGG**TT**GGGG**
	un2	**GGGG**GCGA**GGGG**CGGGA**GGGG**GCGA**GGGG**
	un3	**GGG**A**GG**A**G**C**GGGGGG**A**G**GA**G**C**GGG**
*Taq* polymerase stop assay	un3 template	TTTTTGGGAGGAGCGGGGGGAGGAGCGGGTTTTT*CTGCATATAAGCAGCTGCTTTTTGCC*
	no-G4 template	TTGTCGTTAAAGTCTGACTGCGAGCTCTCAGATC*CTGCATATAAGCAGCTGCTTTTTGCC*
	primer	GGCAAAAAGCAGCTGCTTATATGCAG
qPCR	UL30 primer F	TTCGACTTTGCCAGCCTGTA
	UL30 primer R	CAGGGAGAGCGTGCTGAAG
	actin primer F	TCACTGAGCGCGGCTACA
	actin primer R	CCTTAATGTCACGCACGATTTC
RT-qPCR	ICP34.5 primer F	CGCCTTCTTGTTCGCTGCTG
	ICP34.5 primer R	TCGTCGTCATCGTCGTCGTC
	UL36 primer F	AGGGAGGATGCCCACGAA
	UL36 primer R	TCCGCGTCTTCCACAAATC
	UL30 primer F	CAGGGAGAGCGTGCTGAAG
	UL30 primer R	TTCGACTTTGCCAGCCTGTA
	ICP22 primer F	GGCCCGGAGTGTGATCTTAG
	ICP22 primer R	GGTGGCATCGGAGATTTCAT
	ACTB primer F	CCTTAATGTCACGCACGATTTC
	ACTB primer R	TCACTGAGCGCGGCTACA
	p62 primer F	TGCCCAGACTACGACTTGTG
	p62 primer R	AGTGTCCGTGTTTCACCTTCC

* Gs that may be involved in G4 formation are shown in bold. Bases that anneal to the primer sequence in the template oligonucleotides for *Taq* polymerase stop assay are shown in italics.

## Data Availability

The data presented in this study are available in the article.
